# Condensation of ground state from a supercooled phase in the Si(111)-(4 × 1) → (8 × 2)-indium atomic wire system

**DOI:** 10.1063/1.5111636

**Published:** 2019-08-02

**Authors:** B. Hafke, T. Witte, D. Janoschka, P. Dreher, F.-J. Meyer zu Heringdorf, M. Horn-von Hoegen

**Affiliations:** Department of Physics and CENIDE, University Duisburg-Essen, Lotharstr. 1, 47057 Duisburg, Germany

## Abstract

Strong optical irradiation of indium atomic wires on a Si(111) surface causes the nonthermal structural transition from the (8 × 2) reconstructed ground state to an excited (4 × 1) state. The immediate recovery of the system to the ground state is hindered by an energy barrier for the collective motion of the indium atoms along the reaction coordinate from the (4 × 1) to the (8 × 2) state. This metastable, supercooled state can only recover through nucleation of the ground state at defects like adsorbates or step edges. Subsequently, a recovery front propagates with constant velocity across the surface and the (8 × 2) ground state is reinstated. In a combined femtosecond electron diffraction and photoelectron emission microscopy study, we determined—based on the step morphology—a velocity of this recovery front of ∼100 m/s.

## I. INTRODUCTION

The indium induced (4 × 1) reconstruction on Si(111) is a prototypical atomic wire system of metal atoms on a surface and has been thoroughly investigated since it was first mentioned in 1964.^1,2^ During the last decade, the system has attracted attention, because the indium wires undergo a reversible phase transition from the (4 × 1) high temperature (HT) phase to the (8 × 2) reconstructed low temperature (LT) ground state at a critical temperature of *T_c_* = 120 K. The periodicity doubling along and perpendicular to the wires is explained by a Peierls-like mechanism where more than one electronic band is involved.^3–6^ The structural transition is accompanied by the opening of a bandgap, a metal to insulator transition, and formation of a charge density wave.^3–10^

**FIG. 1.**
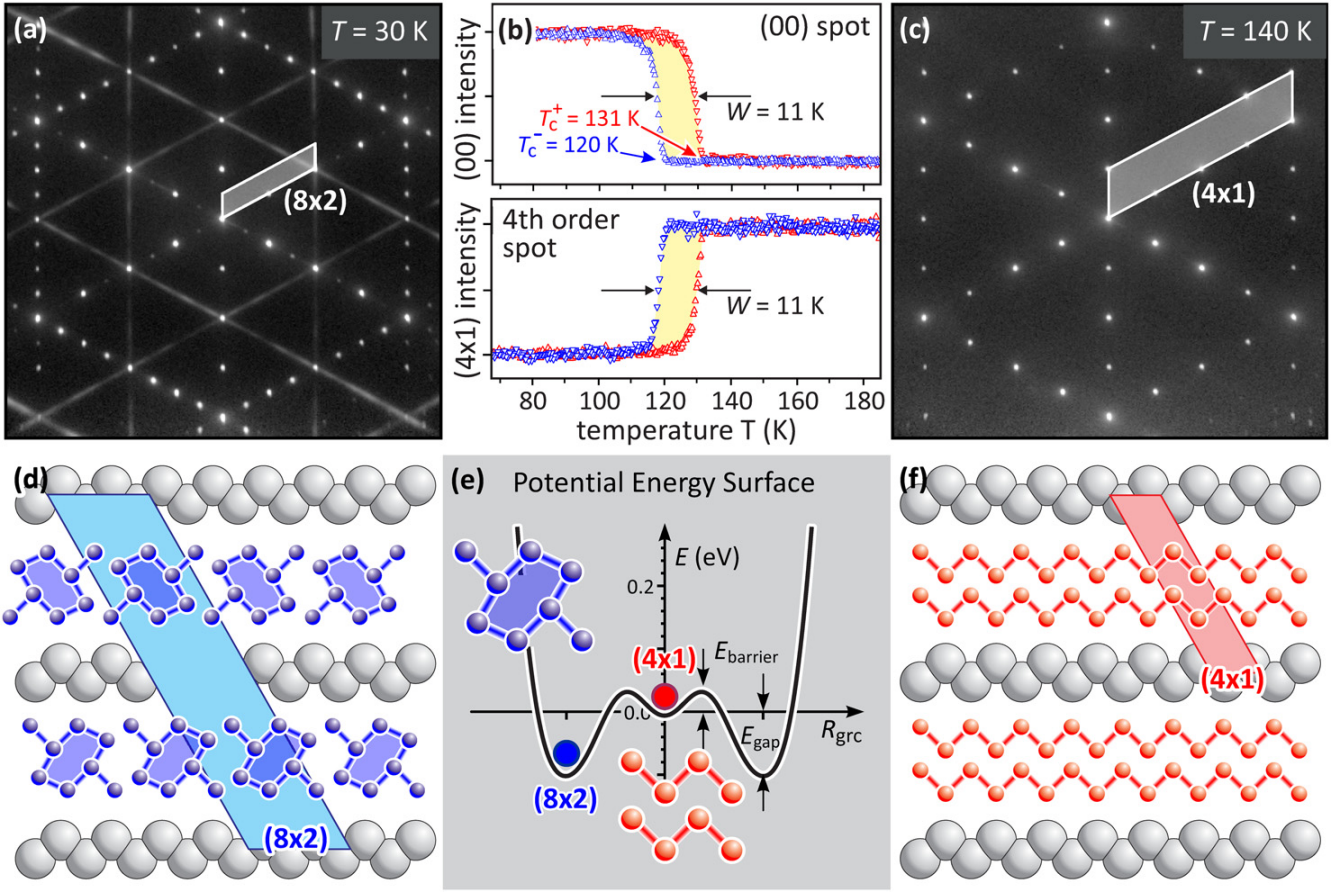
(a) and (c) LEED diffraction patterns of the (8 × 2) LT ground state and the (4 × 1) HT state. The distorted hexagon structure of the indium atoms in the insulating LT state is sketched in blue. The zigzag chain of the indium atoms in the metallic HT state is sketched in red. (b) Hysteretic behavior of the (00) spot and the 4th order spot intensity upon slow temperature cycling. Red and blue symbols indicate heating and cooling, respectively. (d) and (f) Arrangement of indium atoms along the wire direction. Sketch of the distorted hexagons (blue) and zigzag chains (red) of the indium atoms in the insulating (8 × 2) LT and the metallic (4 × 1) HT state, respectively. Gray filled circles indicate the topmost Si atoms. (e) Potential energy surface as a function of the reaction coordinate *R*_grc_ between the two degenerate minima of the (8 × 2) ground state and the high-lying (4 × 1) state. The bandgap *E*_gap_ and energy barrier *E*_barrier_ are indicated.

The HT (4 × 1) structure is composed of indium atoms arranged in zigzag chains [see Figs. 1(c) and 1(f) for the low energy electron diffraction (LEED) pattern and the real space structure of the outermost surface layer]. Upon cooling, the indium atoms rearrange in distorted hexagons, locally creating a (4 × 2) structure within the atomic wire. The nonvanishing interaction between two adjacent wires results in the observed LT (8 × 2) structure [see Figs. 1(a) and 1(d) for the LEED pattern and the real space structure].^11,12^ Figure 1(b) shows that the intensity of the (00) spot decreases at 131 K upon heating and recovers at 120 K upon cooling. The existence of this hysteretic behavior during temperature cycling through the (8 × 2) ↔ (4 × 1) phase transition proves the first order nature of the phase transition.^13^ The (4 × 1) spot exhibits the opposite behavior: its intensity increases upon transition from the LT to the HT state by a factor of two upon heating and decreases again upon cooling. The transfer of intensity from the (8 × 2) spots to the (4 × 1) spots is observed for almost all of the (4 × 1) spots.^14^ Such a rise of intensity on the expense of the (8 × 2) spots can be rationalized by simple kinematic scattering theory: the overall number of diffracted electrons remains constant as the position of the indium atoms during the transition from (8 × 2) to (4 × 1) structure changes by less than 0.1 Å (Ref. 15)—it is thus small compared to the interatomic distances.

The observed hysteresis is direct evidence for the existence of a significant energy barrier between the (8 × 2) ground state and the (4 × 1) HT phase at *T*_c._^16^ Such an energy barrier has also been predicted by density functional theory for low temperatures *T* < *T*_c_. The ground state potential energy surface (PES) shown in Fig. 1(e) exhibits three minima; two of them are the degenerate (8 × 2) LT-ground state *E*_gap_ = 0.1 eV below the (4 × 1) HT state. The HT and LT states are separated by symmetric barriers of *E*_barrier_ = 40 meV.^17^

When irradiated by an IR femtosecond laser pulse, the surface is photoexcited.^17–21^ For sufficiently high incident fluences (Φ_in_ > 2 mJ/cm^2^), the entire surface undergoes a photoinduced transition from the LT to the HT state.^17,18^ It is important to note that the structural transition occurs before an overall heating of the surface can be detected: the transient heating of the surface is delayed by 6 ps (Ref. 22) with an overall temperature rise between Δ*T *=* *15–70 K for incident fluences of Φ_in_ = 2–7 mJ/cm^2^, respectively. Considering the static sample temperature *T*_0_ = 30 K ≪ *T*_c_, the maximum transient surface temperature *T*_max_(6 ps) = (100 ± 10) K at Φ_in_ = 7 mJ/cm^2^ remains below *T*_c_ = 130 K. Thus, the structural transition must be nonthermal and is driven by electronic entropy in less than one picosecond.^19^

The immediate recovery of the system LT (8 × 2) ground state is prevented by the 40 meV barrier between HT and LT phases. The existence of this barrier has also been confirmed in an independent time-resolved electron spectroscopy study.^19^ At 30 K, i.e., with k_B_*T* = 2.5 meV, the barrier cannot be overcome by thermal excitation on the observed time scale. In our previous work, we have shown that adsorbates from the residual gas act as seed for the recovery of the ground state, which is mediated by a 1D recrystallization front that propagates like a row of falling dominoes.^17^ Accordingly, with increasing adsorbate density, the time-constant for recovery of the ground state decreases. From a comparison of the density of adsorbates with a shift of transition temperature, a velocity of the recovery front of *v*_rec_ = 82 m/s was determined.^17^

Here, we present a combined femtosecond electron diffraction and surface microscopy study under vastly improved vacuum conditions. We find that the recovery from the HT excited state to the LT ground state is not determined by adsorbates any more but proceeds through heterogeneous nucleation at step edges. With the added knowledge of the step morphology of the Si(111) substrate, a direct determination of the recovery front's velocity is possible which is independent of the adsorbate density.

## II. EXPERIMENTAL SETUP AND SAMPLE PREPARATION

The experiments were performed under ultrahigh vacuum conditions at a base pressure of p < 10^−10^ mbar. Well oriented Si(111) samples (Virginia Semiconductors, phosphor doped, 0.6–1.0 Ω cm, miscut < 0.1°) were mounted on a liquid Helium cryostat, allowing for sample cooling to a minimum temperature of 30 K. Heating of the cryostat allowed us to adjust the sample temperature between 30 K and 450 K. The sample was heated by direct current heating. Clean Si surfaces were prepared by repeated short flash-anneal cycles up to 1250 °C.

One monolayer (ML, 1 ML = 7.8 × 10^14^ atoms cm^−2^) of indium atoms was deposited *in situ* from a graphite crucible at a sample temperature of 450 °C. Self-assembly of the indium atoms results in the formation of the HT (4 × 1) reconstruction in three equivalent rotational domains. The integrity of the reconstruction prior to deposition, during deposition, and after each experiment was confirmed by low-energy electron diffraction (LEED) and reflection high-energy electron diffraction (RHEED). The step morphology of the samples was determined in a photoelectron emission microscope (PEEM) after *in situ* step decoration through submonolayer deposition of Ag at slightly elevated temperature.

Time-resolved RHEED is employed to follow both the reversible excitation from the ground state to the HT phase and the recovery back to the ground state. We applied a pump-probe scheme in which ultrashort laser pulses excite the sample and 30 keV electron pulses probe the temporal evolution of the diffraction pattern at different time delays Δ*t.*^23,24^ We used 800 nm laser pulses of 80 fs duration from a Ti:Sapphire chirped pulse amplifier with a 5 kHz repetition rate. A tilted pulse front scheme compensated the velocity mismatch at the sample between the electrons (under grazing incidence) and the laser pulses (under normal incidence).^25^ The temporal resolution of the entire experiment is 330 fs (full width at half maximum of the temporal instrumental response function).^26^

All RHEED experiments were performed at a sample base temperature of *T*_0_ = 30 K. Stationary heating of the cooled Si substrate through the incident laser irradiation was less than 4 K. The PEEM micrographs were recorded at room temperature.

## III. RESULTS AND DISCUSSION

**FIG. 2.**
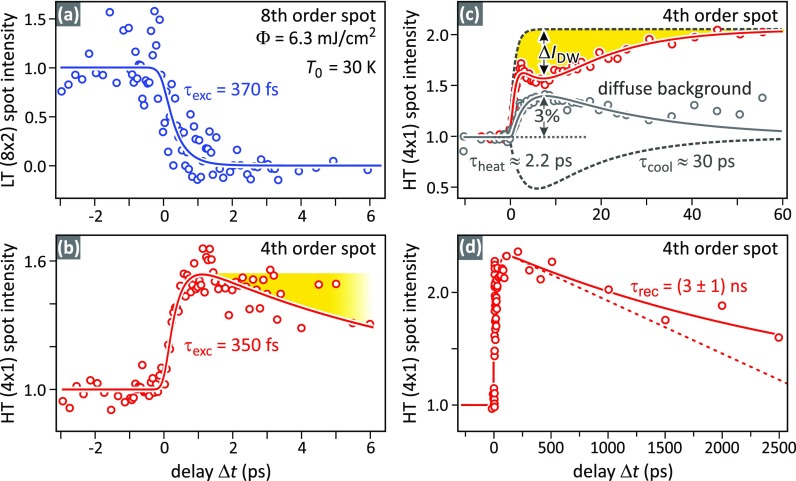
Excitation dynamics and recovery kinetics of the phase transition at a sample temperature of *T*_0_ = 30 K and an incident laser fluence of Φ_in_ = 6.3 mJ/cm^2^. Plotted are RHEED spot intensities as a function of time delay Δ*t.* (a) The drop of the (8 × 2) spot intensity reflects the lifting of the LT state in less than 1 ps. (b) At the same time delay, the (4 × 1) spot intensity rises and indicates the structural phase transition. (c) The transient drop of the intensity of the (4 × 1) spot and the rise of the diffuse background intensity are attributed to the Debye Waller effect and are caused by a delayed onset of the thermal motion of the indium atoms. (d) On very long time scales, τ_rec_ = (3 ± 1) ns, the intensity of the (4 × 1) spot recovers to the value it had prior to the excitation.

The transient intensities of the (8 × 2) and (4 × 1) spots in the RHEED diffraction pattern are analyzed to determine the relative fractions of the surface in the HT and LT phase on a femtosecond time scale. Figures 2(a) and 2(b) depict the changes of the RHEED spot intensity through the first few picoseconds of the structural transition from (8 × 2) LT to (4 × 1) HT state. The transition to the HT-phase on a subpicosecond time scale is reflected in the disappearance of all (8 × 2) spots. On the same time scale, complementing this behavior, the (4 × 1) spot intensity increases by a factor of two. Figure 2(c) shows a transient drop of (4 × 1) spot intensity with a minimum at 6 ps time delay which is caused by the Debye-Waller effect and is due to delayed heating of the surface.^22^

The excited HT phase survives up to (3 ± 1) ns as is evident from the rather slow drop-off of the (4 × 1) spot intensity, which is shown in Fig. 2(d). We explain this slow recovery of the LT ground state through a 1D recovery front along the indium wires starting from pre-existing seeds.^17^ Such seeds can be morphological defects like persistent contaminations or steps at the surface. Since we do not observe large changes of the recovery time constant τ_rec_ for hours after sample preparation, we can safely rule out homogenous seeds like adsorbates from the residual gas. We are thus able to determine a maximum value for the velocity of the 1D recrystallization front, assuming that the only relevant seeds are steps at the surface.

Let us assume that the recovery front starts at every step edge and propagates with (constant) average velocity *v*_rec_ across either the upper or the lower terrace. Considering an isolated, single terrace with constant terrace width Γ would then produce a linear change of diffracted intensities. More precisely, we would expect a linear increase in the (8 × 2) intensity and a linear decrease in the (4 × 1) intensity to their initial values. The time *t* = Γ/*v*_rec_ needed to convert the terrace from the metastable excited (4 × 1) HT state to the (8 × 2) LT ground state is determined by the terrace width Γ and the recovery velocity *v*_rec_. In the real system, a statistical distribution of terrace widths Γ_*i*_ will produce individual recovery times *t_i_*, which must be incoherently superposed in the diffraction experiment where the superposition results in a nonlinear recovery of intensity with time. Thus, to interpret the temporal behavior and to estimate the velocity of recovery front, we first need to determine the terrace widths Γ_*i*_ of the surface.

**FIG. 3.**
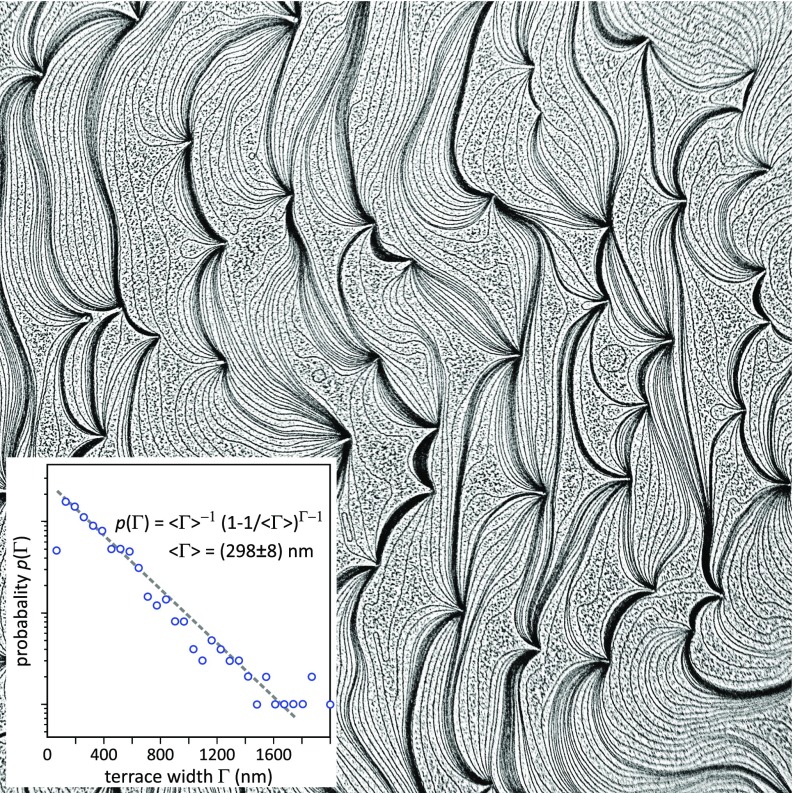
PEEM image of Ag-decorated Si(111) with a field of view 50 × 50 *μ*m^2^. Steps are represented as dark lines. The image is contrast inverted for better visibility. Dark lines and clusters correspond to Ag agglomerates with a locally lowered work function. The step down direction is from left to right. The inset depicts the terrace width distribution *p*(Γ) on a logarithmic scale.

The step density and terrace width of our Si(111) sample were determined with PEEM. The PEEM micrograph in Fig. 3 shows a representative area of the surface with a field of view of 50 × 50 *μ*m^2^. Ag decoration was used to emphasize the steps, and due to slight changes of the local work function from areas covered with and without Ag, the steps appear dark in Fig. 3 (note that the image was contrast-inverted for better visibility). The surface of the used Si(111) substrate exhibits 200 ± 40 steps within the field of view, i.e., a low step density of 4 ± 0.8 *μ*m^−1^. This step density corresponds to a misorientation of the sample of 0.075° ± 0.015° with respect to the (111) orientation. The overall step morphology is typical for a Si(111) surface, the used preparation technique, and the large field of view. Many faint dark lines (single-height decorated Si steps) are visible on the surface; they coalesce at defect locations and form step-bunches between adjacent defects. This step morphology is formed during the initial high-temperature flash-annealing to *T *>* *1250 °C that removes the native silicon oxide layer from the sample and leaves behind a low density of defects, such as silicon carbide contaminations. At the high flash-annealing temperatures, silicon sublimates from the surface and this desorption of material is reflected in a retraction of the steps in the step-up direction. As the step-movement is pinned at the defects, the steps become bunched. Between the pinning centers, the steps are free to move, but due to the boundary conditions and the step-stiffness, their energetically favorable shape is convex in the step-up direction. We thus conclude that the step-up direction is from right to left.

Step bunches where the individual steps are so close to each other that they cannot be distinguished contain narrow terraces of widths Γ_*i*_ < 50 nm. These step bunches cover an area ∼10% of the surface as estimated from image filtering with contrast enhancement. We therefore excluded these areas for the determination of the terrace width probability *p*(Γ) which is defined as the probability to find a terrace of width Γ. This distribution *p*(Γ) is plotted in the inset of Fig. 3 for terraces wider than Γ_*i*_ > 50 nm. The value for the narrowest terraces is flawed because of the limited resolution of the PEEM. Apart from that, the terrace width distribution can be fitted well by a power law behavior *p*(Γ) = ⟨Γ⟩^−1^ (1 − ⟨Γ⟩^−1^)^(Γ−1)^, indicating a geometric terrace width distribution with a mean value ⟨Γ⟩ = (298 ± 8) nm.

**FIG. 4.**
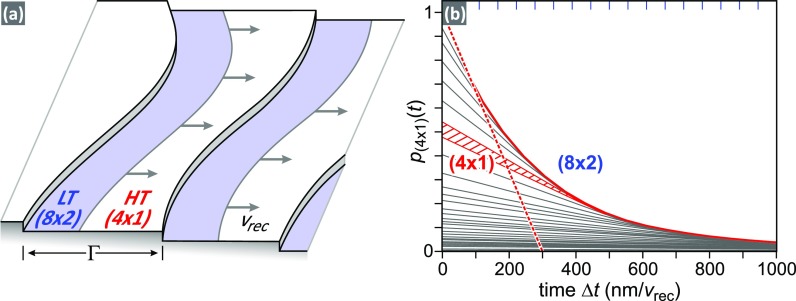
(a) Sketch of the stepped Si(111) surface with terrace width Γ. The LT (8 × 2) state (light blue) propagates with velocity *v*_rec_ from the step edge and reduces the area covered with the metastable HT (4 × 1) excited state. (b) Modeled fraction of the surface that is still in the HT (4 × 1) state plotted as a function of time delay Δ*t*. The red dashed line depicts the initial slope *v*_rec_/298 nm of the recovery of the ground state as a function of time. The simulation is based on the terrace width distribution from Fig. 3.

For further analysis, we assume a propagation of the recovery front with constant mean velocity *v*_rec,_ as sketched in Fig. 4(a). Based on the terrace width distribution *p*(Γ), the temporal response of the system can be modeled. The time *t_i_* = Γ_*i*_/*v*_rec_ is required for the complete recovery of a terrace of width Γ_*i*_ to the ground state. The fraction of a terrace that already recovered the ground state can be denoted as a function of time as

$$p_{\Gamma }(t\leq t_{i})=t\cdot v_{\text{rec}}/\Gamma_{i}\;\text{and}\;p_{\Gamma }\left(t>t_{i}\right)=1.$$

An example for the recovery of terraces with a width 450 < Γ_*i*_ < 520 nm is shown as a red shaded triangle in Fig. 4(b). The height of the triangle reflects the area of the terraces which are still in the (4 × 1) HT state as a function of time delay Δ*t*. The linear recovery starts at Δ*t *=* *0 and ends at Δ*t *=* *440 nm/*v*_rec_, when the conversion of the (4 × 1) HT to the (8 × 2) LT state on these terraces is completed.

The fraction of the surface *p*_(4 × 1)_(*t*) which has not yet recovered the (8 × 2) LT ground state is then given by

$$p_{\left(4\times 1\right)}\left(t\right)=1-\int_{0}^{\Gamma_{\text{max}}}p_{\Gamma }\left(t\right)\cdot p\left(\Gamma \right)\cdot \Gamma \;d\Gamma ,$$

with Γ_max_ being the maximum observed terrace width.

The resulting *p*_(4 × 1)_(*t*) is shown as a solid red line in Fig. 4(b) as a function of Δ*t* in units of nanometers per *v*_rec_. It resembles an exponential decay. The slope for short time delays is indicated by the dashed red line, which yields a recovery velocity of *v*_rec_/298 nm. Comparing this value to the decay time constant τ_rec_ = (3 ± 1) ns of the (4 × 1) intensity that we obtained from the dashed line in Fig. 2(d), we conclude a maximum velocity of the recovery front *v*_rec_ = (100 ± 40) m/s.

## IV. CONCLUSIONS

Time resolved electron diffraction and photoelectron emission microscopy were used to study the recovery kinetics from a metastable supercooled state to the ground state in the Si(111)–(8 × 2)-In atomic wire system. This system exhibits a first order structural phase transition at *T*_c_ = 130 K when it is converted to a high symmetry (4 × 1) phase. This high temperature phase can also be obtained by femtosecond-laser irradiation at low temperatures *T*_0_ ≪ *T*_c_. A small energy barrier along the reaction coordinate from the (4 × 1) to the (8 × 2) phase hinders the immediate recovery to the (8 × 2) ground state and for nanoseconds leaves the system in a metastable supercooled state.

The recovery of the ground state is facilitated through nucleation at pre-existing step edges of the Si substrate. The step edges act as heterogeneous seeds for the recovery front which propagates like a row of falling dominoes at a speed of *v*_rec_ ∼100 m/s across the terraces of the slightly miscut Si(111) surface. The measured speed is by almost one order of magnitude lower than that predicted through a molecular dynamics simulation.^17^ This may be explained by the strictly one-dimensional approach in the simulation, which does not take the 2D correlation of adjacent wires into account.

Since the recovery time is found to depend on the terrace-width distribution, our study demands future experiments on Si surfaces with a higher miscut angle. It is known that misorientations of the order of 1° relative to the (111) orientation yield narrower terrace width distributions, i.e., more ordered step-trains. Such surfaces show only one of the three rotational (8 × 2) domains^19^ and exhibit straight step edges.^27^ On these narrower terraces, the recovery time constant will be faster, i.e., of the order of a few 100 ps—which is a regime of time delay that can be accessed more easily in our experiment. Such surfaces will thus be ideal templates to explore the influence of temperature, excitation density, or supercooling on the speed of the recovery front.

## References

[1] J. J. Lander and J. Morrison , “ Surface reactions of silicon with aluminium and with indium,” Surf. Sci. 2, 553 (1964). 10.1016/0039-6028(64)90099-8

[2] P. C. Snijders and H. H. Weitering , “ Electronic instabilities in self-assembled atom wires,” Rev. Mod. Phys. 82, 307 (2010). 10.1103/RevModPhys.82.307

[3] H. W. Yeom , S. Takeda , E. Rotenberg , I. Matsuda , K. Horikoshi , J. Schaefer , C. M. Lee , S. D. Kevan , T. Ohta , T. Nagao , and S. Hasegawa , “ Instability and charge density wave of metallic quantum chains on a silicon surface,” Phys. Rev. Lett. 82, 4898 (1999). 10.1103/PhysRevLett.82.4898

[4] O. Gallus , T. Pillo , M. Hengsberger , P. Segovia , and Y. Baer , “ A system with a complex phase transition: Indium chains on Si(111),” Eur. Phys. J. B 20, 313 (2001). 10.1007/BF01352592

[5] J. R. Ahn , J. H. Byun , H. Koh , E. Rotenberg , S. D. Kevan , and H. W. Yeom , “ Mechanism of gap opening in a triple-band Peierls system: In atomic wires on Si,” Phys. Rev. Lett. 93, 106401 (2004). 10.1103/PhysRevLett.93.106401 15447426

[6] Y. J. Sun , S. Agario , S. Souma , K. Sugawara , Y. Tago , T. Sato , and T. Takahashi , “ Cooperative structural and Peierls transition of indium chains on Si(111),” Phys. Rev. B 77, 125115 (2008). 10.1103/PhysRevB.77.125115

[7] S. J. Park , H. W. Yeom , S. H. Min , D. H. Park , and I.-W. Lyo , “ Direct evidence of the charge ordered phase transition of indium nanowires on Si (111),” Phys. Rev. Lett. 93, 106402 (2004). 10.1103/PhysRevLett.93.106402 15447427

[8] G. Lee , J. Guo , and E. W. Plummer , “ Real-space observation of nanoscale inhomogeneities and fluctuations in a phase transition of a surface quasi-one-dimensional system: In/Si(111),” Phys. Rev. Lett. 95, 116103 (2005). 10.1103/PhysRevLett.95.116103 16197023

[9] J. Guo , G. Lee , and E. W. Plummer , “ Intertwined electronic and structural phase transitions in the In/Si(111) interface,” Phys. Rev. Lett. 95, 046102 (2005). 10.1103/PhysRevLett.95.046102 16090824

[10] E. Speiser , S. Chandola , K. Hinrichs , M. Gensch , C. Cobet , S. Wippermann , W. G. Schmidt , F. Bechstedt , W. Richter , K. Fleischer *et al.*, “ Metal-insulator transition in Si(111)-(4 × 1)/(8 × 2)-In studied by optical spectroscopy,” Phys. Status Solidi B 247, 2033 (2010). 10.1002/pssb.200983961

[11] S. Wippermann and W. G. Schmidt , “ Entropy explains metal-insulator transition of the Si(111)-In nanowire array,” Phys. Rev. Lett. 105, 126102 (2010). 10.1103/PhysRevLett.105.126102 20867660

[12] W. G. Schmidt , S. Wippermann , S. Sanna , M. Babilon , N. J. Vollmers , and U. Gerstmann , “ In-Si(111)(4 × 1)/(8 × 2) nanowires: Electron transport, entropy, and metal insulator transition,” Phys. Status Solidi B 249, 343 (2012). 10.1002/pssb.201100457

[13] F. Klasing , T. Frigge , B. Hafke , S. Wall , B. Krenzer , A. Hanisch-Blicharski , and M. Horn-von Hoegen , “ Hysteresis proves that the In/Si(111) (8 × 2) to (4 × 1) phase transition is first-order,” Phys. Rev. B 89, 121107 (2014). 10.1103/PhysRevB.89.121107

[14] M. Horn-von Hoegen , “ Ultrafast switching in an atomic wire system at surfaces: A time-resolved reflection high-energy electron diffraction study,” MRS Bull. 43, 512 (2018). 10.1557/mrs.2018.150

[15] W. G. Schmidt , private communication (2017).

[16] L. Landau and E. Lifschitz , *Statistical Physics* ( Pergamon Press, New York, 1951).

[17] S. Wall , B. Krenzer , S. Wippermann , S. Sanna , F. Klasing , A. Hanisch-Blicharski , M. Kammler , W. G. Schmidt , and M. Horn-von Hoegen , “ Atomistic picture of charge density wave formation at surfaces,” Phys. Rev. Lett. 109, 186101 (2012). 10.1103/PhysRevLett.109.186101 23215299

[18] T. Frigge , B. Hafke , T. Witte , B. Krenzer , C. Streubühr , A. Samad Syed , V. Mikšić Trontl , I. Avigo , P. Zhou , M. Ligges , D. von der Linde , U. Bovensiepen , M. Horn-von Hoegen , S. Wippermann , A. Lücke , S. Sanna , U. Gerstmann , and W. G. Schmidt , “ Optically excited structural transition in atomic wires on surfaces at the quantum limit,” Nature 544, 207 (2017). 10.1038/nature21432 28355177

[19] C. W. Nicholson , A. Lücke , W. G. Schmidt , M. Puppin , L. Rettig , R. Ernstorfer , and M. Wolf , “ Beyond the molecular movie: Dynamics of bands and bonds during photoinduced phase transition,” Science 362, 821 (2018). 10.1126/science.aar4183 30442808

[20] M. Chávez-Cervantes , R. Krause , S. Aeschlimann , and I. Gierz , “ Band structure dynamics in indium wires,” Phys. Rev. B 97, 201401(R) (2018). 10.1103/PhysRevB.97.201401

[21] C. W. Nicholson , M. Puppin , A. Lücke , U. Gerstmann , M. Krenz , W. G. Schmidt , L. Rettig , R. Ernstorfer , and M. Wolf , “ Excited-state band mapping and momentum-resolved ultrafast population dynamics in In/Si (111) nanowires investigated with XUV-based time- and angle-resolved photoemission spectroscopy,” Phys. Rev. B 99, 155107 (2019). 10.1103/PhysRevB.99.155107

[22] T. Frigge , B. Hafke , T. Witte , B. Krenzer , and M. Horn-von Hoegen , “ Non-equilibrium lattice dynamics of one-dimensional In chains on Si (111) upon ultrafast optical excitation,” Struct. Dyn. 5, 025101 (2018). 10.1063/1.5016619 29607349 PMC5869048

[23] A. Janzen , B. Krenzer , O. Heinz , P. Zhou , D. Thien , A. Hanisch , F.-J. Meyer zu Heringdorf , D. von der Linde , and M. Horn-von Hoegen , “ A pulsed electron gun for ultrafast electron diffraction at surfaces,” Rev. Sci. Instrum. 78, 13906 (2007). 10.1063/1.2431088 17503932

[24] A. Janzen , B. Krenzer , P. Zhou , D. von der Linde , and M. Horn-von Hoegen , “ Ultrafast electron diffraction at surfaces after laser excitation,” Surf. Sci. 600, 4094 (2006). 10.1016/j.susc.2006.02.070 17503932

[25] P. Zhou , C. Streubühr , A. Kalus , T. Frigge , S. Wall , A. Hanisch-Blicharski , M. Kammler , M. Ligges , U. Bovensiepen , D. von der Linde , and M. Horn-von Hoegen , “ Ultrafast time resolved reflection high energy electron diffraction with tilted pump pulse fronts,” EPJ Web Conf. 41, 10016 (2013). 10.1051/epjconf/20134110016

[26] B. Hafke , T. Witte , C. Brand , T. Duden , and M. Horn-von Hoegen , “ Pulsed electron gun for electron diffraction at surfaces with femtosecond temporal resolution and high coherence length,” Rev. Sci. Instrum. 90, 045119 (2019). 10.1063/1.5086124 31042971

[27] J. Viernow , J.-L. Lin , D. Y. Petrovykh , F. M. Leibsle , F. K. Men , and F. J. Himpsel , “ Regular step arrays on silicon,” Appl. Phys. Lett. 72, 948 (1998). 10.1063/1.120882

